# Intensive care due to myasthenia gravis: Risk factors and prognosis

**DOI:** 10.1111/ene.16522

**Published:** 2024-10-22

**Authors:** Chris Myllynen, Anni Tuulasvaara, Sari Atula, Sini M. Laakso

**Affiliations:** ^1^ Department of Neurosciences University of Helsinki Helsinki Finland; ^2^ Department of Neurology, Brain Center Helsinki University Hospital Helsinki Finland; ^3^ Translational Immunology Research Program University of Helsinki Helsinki Finland

**Keywords:** exacerbation, intensive care, myasthenia gravis, myasthenic crisis, risk factors

## Abstract

**Background and purpose:**

Exacerbation of myasthenia gravis (MG) with respiratory failure requires intensive care. We aimed to study the risk factors for intensive care unit admission for MG exacerbation and myasthenic crisis (MC) and the prognosis of people with MG (pwMG) thereafter.

**Methods:**

This retrospective study investigated patients in the Helsinki and Uusimaa hospital district during the years 2008–2021. PwMG (International Classification of Diseases, 10^th^ revision code G70.0) were identified through a data repository search, followed by a chart review of patient records. Risk factors for intensive care due to MG exacerbation were evaluated as compared with the patients only treated in the outpatient clinic and those treated in the neurological ward for MG exacerbation. The outcomes of patients in intensive care for any reason were also compared with those of patients in intensive care for exacerbation of *bronchial asthma*.

**Results:**

Of 577 pwMG, 35 (6.1%) needed intensive care for MG within a median of 5.3 months from diagnosis. The mean (±SD) age at MG diagnosis was higher in the intensive care group (60.5 [±16.1] years) compared to the outpatient (48.3 [±20.9] years; *p* < 0.001) and neurological ward groups (53.4 [±20.8] years; *p* = 0.044). Thymoma (odds ratio [OR] 4.8, 95% confidence interval [CI] 1.19–19.43; *p* = 0.028) and female sex (OR 2.1, 95% CI 1.02–4.48; *p* = 0.045) were independent risk factors for intensive care. In‐hospital mortality was 4% for MC patients. Six‐month mortality after intensive care for MG exacerbation (14.3%) was twice that for asthma exacerbation (7.7%).

**Conclusion:**

Our study shows an increased risk of intensive care treatment for patients with late‐onset MG, female sex or thymoma, occurring usually within 6 months from diagnosis, which emphasises the importance of early treatment choices.

## INTRODUCTION

Myasthenia gravis (MG) is an autoimmune disease in which autoantibodies (Abs) disrupt neuromuscular transmission, usually targeting acetylcholine receptors (AChRs) [1, 2]. MG can be divided into subgroups based on onset age, autoantibody status, affected muscles, and thymus pathology [3]. Symptoms range from diplopia and ptosis in ocular MG to weakness of limbs, bulbar or respiratory muscles (generalised MG) [1, 2]. The course of MG varies; complete remission is achieved by 22% of AChR‐Ab‐positive patients in a mean of 6 years from diagnosis, but 15%–20% of patients experience myasthenic crisis (MC), a severe exacerbation with acute respiratory failure [4, 5, 6, 7]. The prevalence of MG ranges from 5.4 to 35 per 100,000 worldwide and is 20.9 per 100,000 in Finland [8].

Many factors can induce MG exacerbations. Infections, especially respiratory, are the most common trigger [5, 9]. Other factors include surgery, pregnancy, stress, thyroid dysfunction, inadequate MG treatment and medications [6, 9, 10, 11, 12]. Low serum potassium is speculated to worsen MG by disrupting muscle–nerve junction function [13]. In 20%–40% of MC no precipitating factor can be identified [6, 9, 14]. Patient‐specific risk factors for MG exacerbation and hospitalisation include disease onset after the age of 50 years (late‐onset MG [LOMG]), comorbidities, previous MC, Abs against muscle‐specific kinase (MuSK), thymoma and severe disease at diagnosis [14, 15, 16]. Conversely, Myasthenia Gravis Foundation of America (MGFA) minimal manifestation status after 12 months of MG diagnosis indicates lower exacerbation risk [14, 17].

*Bronchial asthma* is another autoimmune disease sometimes requiring intensive care with mechanical ventilation. The risk factors for intensive care for asthma exacerbation partially overlap with those of MG, including infections [18].

Due to therapeutic advances, mortality in MC has decreased to 5%–12% in recent decades [6, 7, 19, 20]. Nevertheless, the understanding of prognostic factors for risk and outcome of severe exacerbations of MG is incomplete, and mortality among MG patients is increasing in the Nordic countries [8]. Our study addresses the need for intensive care of people with MG (pwMG) for all reasons in our hospital district, and analyses the risk factors, features, and prognosis of MG exacerbation and MC requiring intensive care. We compare pwMG in intensive care to those admitted to regular hospital wards, those only in outpatient follow‐up, and patients requiring intensive care for *bronchial asthma*.

## MATERIALS AND METHODS

### Study design and cohort

The patient cohort for this retrospective registry study was derived from the data repository data lake of the Hospital District of Helsinki and Uusimaa (HUS), Finland [21], in collaboration with the HUS Data Service. The research permit for the study was obtained from the Helsinki University Hospital (HUS/313/2022). According to Finnish law, ethics committee approval was not required as the study used only medical record data.

**FIGURE 1:**
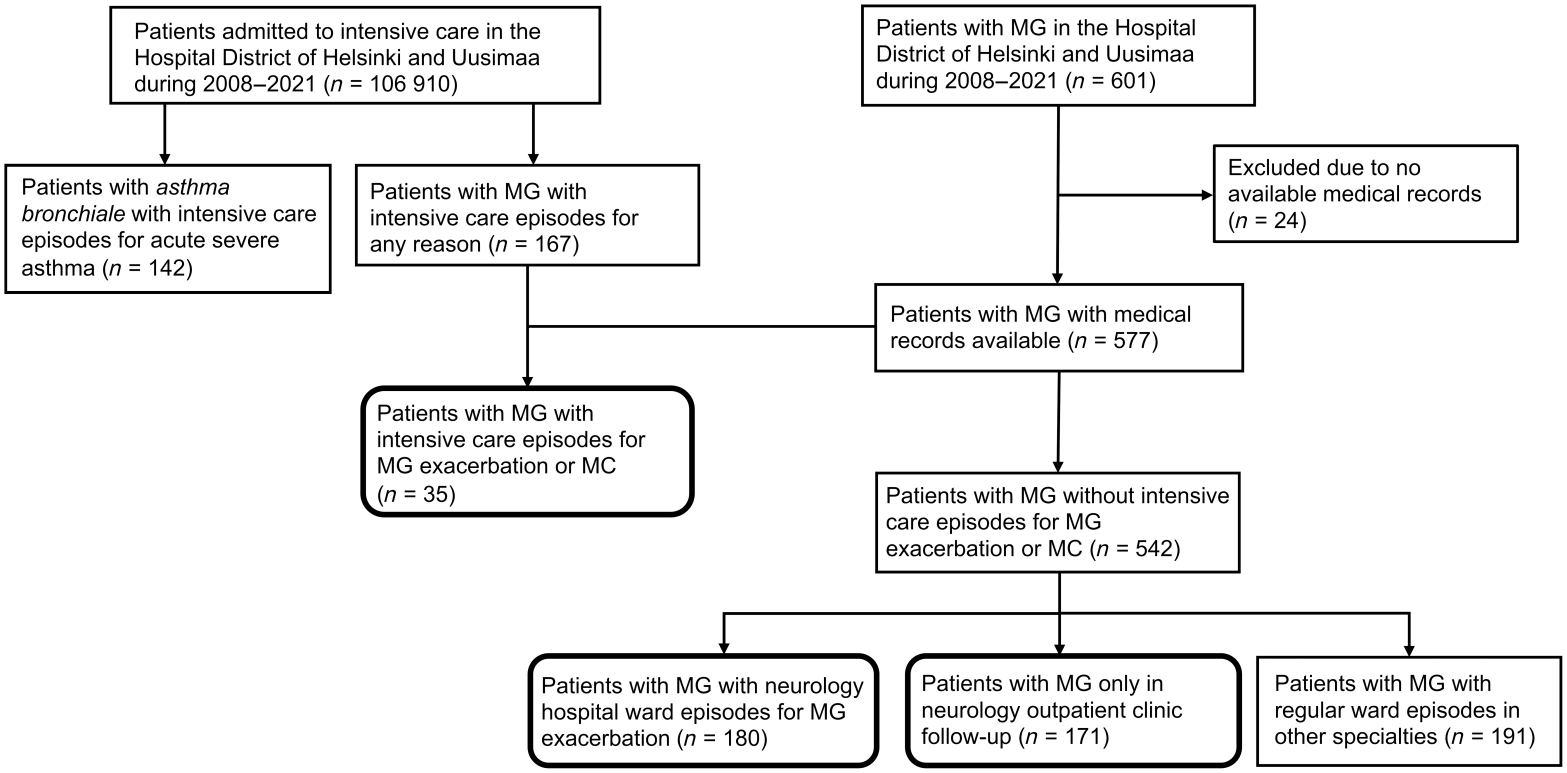
Study subgroups. Flow chart showing data acquisition and formation of the study subgroups. The main subgroups of the study are highlighted. There was partial overlap between people with myasthenia gravis (pwMG) in intensive care for reasons other than myasthenia gravis (MG) and hospital ward groups; some pwMG in intensive care for MG also had episodes in regular or neurological wards. The total number 577 of pwMG included the intensive care group, neurological ward group, outpatient care group, and patients in regular wards, to exclude duplicates. In intensive care for other reasons were 99 of 167 pwMG who did not come up in other groups, and they were excluded from the comparisons due to the possibility of MG affecting the intensive care admission. MC, myasthenic crisis.

A search was conducted for patients with intervention codes for intensive care or intensive‐monitoring ward care from 1 January 2008 to 31 December 2021, the period of comprehensive data in the data lake, across all regional hospitals and Helsinki University Hospital in the HUS area (Figure 1). Patients with International Classification of Diseases, 10^th^ revision (ICD‐10) code G70.0 (MG) in the medical records at least twice during this period were identified from the patient group with intensive care or intensive‐monitoring ward care, and the patient group with no intensive care codes. Data on intensive care for acute exacerbation of *bronchial asthma* (ICD‐10 code J46) were also searched. The clinical data were supplemented by manually reviewing electronic medical records. MG diagnosis was confirmed from the medical records based on characteristic clinical examination, AChR‐ab and MuSk‐ab findings, and repetitive nerve stimulation in electroneuromyography. The latest data review was in November 2023.

The PwMG in this study were divided into three subgroups: intensive care; neurological ward care; and outpatient care (Figure 1). Patients in the intensive care group had one or more episodes in intensive care or in an intensive‐monitoring ward due to MC or severe MG exacerbation, diagnosed by a neurologist. MC was defined as worsening of MG requiring intubation, non‐invasive ventilation or nasogastric tube [22]. MG exacerbation was a worsening of MG necessitating in‐hospital treatment due to general muscular or bulbar weakness. All the units included in the intensive care group had 24‐h bedside nurse supervision, patient monitoring and respiratory support. The neurological ward group included patients with one or more episodes in the neurological ward due to MG exacerbation. The outpatient group included patients who never required in‐hospital care due to MG. After reviewing the medical records, duplicates and patients who were in the intensive care MG group for non‐MG‐related reasons were removed.

Asthma patients with one or more episodes in the intensive care or in the intensive‐monitoring ward due to severe exacerbation of asthma were included.

### Data acquisition of clinical and prognostic parameters

Data on age, sex, episode duration, and mortality for all patients with intensive care and for all *bronchial asthma* intensive care patients were collected. The following information on the pwMG were collected: sex; MG subtype; onset age; presence of thymoma; class of symptoms at MG onset (ocular, bulbar or generalised); presence of AChR‐abs and MuSk‐abs; comorbidities; MG medication; prior intensive care admissions; administered in‐hospital treatments; and thymectomy. A diagnosis age of 50 years was defined as the cut‐off for early‐onset MG (EOMG) and LOMG. We used the Charlson Comorbidity Index (CCI) to assess general disease burden and the impact of comorbidities in the patients with need for intensive care unit admission for MG, calculating CCI scores across all three subgroups [23, 24].

We collected clinical data from the first intensive care admission for MG exacerbation or MC, from the last healthcare contact before admission to 6 months after the intensive care. The time from MG diagnosis, precipitating factors, MG treatments, and intensive care rescue therapies, including high‐dose intravenous methylprednisolone, plasma exchange, immunoadsorption, or intravenous immunoglobulin, were recorded. The highest C‐reactive protein (CRP) measurement during hospitalisation, and serum potassium level at admission were determined. We collected the latest follow‐up serum potassium levels of all pwMG not in the intensive care group (neurological ward and outpatient groups), and of all pwMG with episodes in the regular wards (non‐intensive care). We separately collected data from the neurological ward group at the beginning of the hospital ward episode. The serum potassium levels were those obtained from each admission period, while other parameters were derived from the first MG intensive care episode for each patient.

### Outcomes

Clinical outcome measures for MG prognosis included the number and duration of intensive care stays, mortality, and disability measured using the modified Rankin Scale (mRS) and disease severity based on MGFA clinical classification. The mRS, a disability scale with scores ranging from 0 (no symptoms) to 6 (death) [25], was determined retrospectively from the medical records at the last healthcare contact before intensive care admission, after hospitalisation, and 6 months afterwards. MGFA classification ranges from I to V, where class I indicates only ocular weakness, II mild weakness, III moderate weakness, IV severe weakness, and V intubation with or without mechanical ventilation. MGFA class was determined retrospectively from medical records at four time points: at the last healthcare contact before intensive care admission; at admission; while MGFA class was worst in the intensive care unit; and at the next neurology outpatient follow‐up after hospitalisation [17].

### Statistical analysis

Data for normally distributed variables are reported as means and standard deviations (SD), and as medians with interquartile range (IQR) for non‐normally distributed variables. Shapiro–Wilk and Kolmogorov–Smirnov tests were used to assess continuous variable distribution. Categorical variables are summarised with frequencies and percentages, and were analysed using chi‐squared or Fisher's exact tests. Continuous variables were compared using the *t*‐test for normal distributions and Mann–Whitney *U*‐test for non‐normal distribution. Spearman's correlation and logistic and linear regression models were used to predict the relationship between studied risk factors and dependent outcome variables, with odds ratios (ORs) and their 95% confidence intervals (CIs) estimated for each factor. A *p* value of <0.05 was taken to indicate statistical significance. All analyses were performed using SPSS version 29 (IBM, New York, NY, USA).

## RESULTS

### Need for intensive care in patients with MG

The data lake search identified 577 pwMG with medical records available. In total, 167 pwMG had intensive care admission for any reason, of whom 35 patients (6.1%) had this for MG exacerbation or MC (intensive care group). Of the 35 pwMG in the intensive care group, 25 (71.4%) had MC according to our definition, representing 4.3% of all identified pwMG, and 10 pwMG (28.6%) were treated for MG exacerbation. Of the 371 pwMG with in‐hospital episodes in regular wards, 180 patients (31.2%) had MG exacerbation (neurological ward group). The number of pwMG who were only in outpatient follow‐up and never in need of hospitalisation for MG was 171 (29.6%; outpatient care group). The clinical and demographic characteristics of these study groups (Figure 1) are shown in Table 1.

**TABLE 1:** Demographic characteristics of study subgroups.

Characteristic	Intensive care group (due to MG exacerbation or MC)	Patients in intensive care due to MC	Neurological ward group	*p* value	*p* value	Outpatient care group	*p* value	*p* value
*N* (% of the total group of 577 patients with MG)	35 (6.1)	25 (4.3)	180 (31.2)	‐	‐	171 (29.6)	‐	–
Female, *n* (%)	20 (57.1)	13 (52.0)	84 (46.7)	NS	NS	95 (55.6)	NS	NS
Mean (±SD) age at diagnosis of MG, years	60.5 (±16.1)	60.8 (± 16.7)	53.4 (±20.8)	0.044	NS	48.3 (±20.9)	<0.001	0.004
LOMG, *n* (%)	27 (77.1)	20 (80)	105 (58.3)	NS	0.037	88 (51.5)	0.018	0.007
AChR antibodies, *n* (% of cases with available antibody data)	24 (82.8)	17 (81.0)	129 (80.6)	NS	NS	98 (81.7)	NS	NS
Thymectomy performed, *n* (%)	21 (60.0)	15 (60)	112 (62.2)	NS	NS	77 (55.0)	NS	NS
Mean (±SD) age at thymectomy, years	54.7 (±15.7)	54.2 (±19.4)	48.2 (±19.5)	NS	NS	39.9 (±18.1)	0.002	0.009
Thymoma, *n* (%)	4 (11.4)	4 (16.0)	4 (2.2)	0.026	<0.001	2 (1.2)	0.002	<0.001
MG with any comorbidity, *n* (%)	30 (85.7)	21 (84.0)	154 (85.6)	NS	NS	113 (66.1)	<0.001	NS

*Note*: *p*‐values for significant differences are presented between the intensive care group and the neurological ward and outpatient subgroups (first column), as well as separately between patients in intensive care due to MC and the neurological ward and outpatient subgroups (second column).

Abbreviations: AChR, acetylcholine receptor; MC, myasthenic crisis; MG, myasthenia gravis; LOMG, late‐onset myasthenia gravis; NS, nonsignificant.

The most common reason for intensive care in pwMG was MG exacerbation including MC, but most of the intensive care episodes of pwMG were for other reasons. Other frequent reasons included cardiovascular, infectious and respiratory diseases, and surgical and traumatic causes. Distribution of all causes for intensive care episodes in pwMG are shown in Table 2.

**TABLE 2:** Main reasons for intensive care unit admission in patients with myasthenia gravis.

Reason for intensive care	Event count (%)
Myasthenia gravis exacerbation or myasthenic crisis	59 (28.1)
Cardiovascular disease	55 (26.2)
Infection and respiratory diseases	30 (14.3)
Surgical or traumatic causes	19 (9.0)
Gastro‐intestinal	10 (4.8)
Malignancy	10 (4.8)
Renal failure	4 (1.9)
Endocrinology	3 (1.4)
Other neurological disease	2 (1.0)

*Note*: The main reason for intensive care admission was used. For patients with myasthenia gravis (MG) as the main diagnosis code for intensive care admission, patient data was reviewed, and if the patient had an MG exacerbation, it was used as the main reason, regardless of any contributing or triggering factors.

Between 2008 and 2021, the hospital district admitted 106,910 patients into intensive care, recording a total of 154,840 episodes, including brief episodes for medical procedures. The 167 pwMG had altogether 210 episodes, of which 59 episodes (28.1%) in 35 patients were due to MG exacerbations and MC; these accounted for 0.04% of the hospital district intensive care periods. None was referred from outside our hospital district. Altogether 142 *bronchial asthma* patients in intensive care due to asthma exacerbation had 202 episodes, covering 0.13% of all the need for intensive care.

### Risk factors for intensive care due to MG exacerbation

We compared the clinical and demographic characteristics of pwMG in the intensive care group, neurological ward group, and outpatient group, and also of the subgroup with MC (Table 1). Patients with episodes in other than neurological wards were excluded from the risk factor comparison, to rule out a possible confounding effect of MG on hospitalisation.

Patients in the intensive care group were older at MG diagnosis and more often had LOMG compared to the neurological or outpatient groups. Thymectomy was performed at an older age in the intensive care group than in the neurological ward or outpatient groups (54.7, 48.2 and 39.9 years) but with shorter delay (median [IQR] 0.6 [0.8], 0.8 [0.5] and 1.0 [1.0] years, respectively; *p* = 0.03). All patients with thymoma in the intensive care group met the MC criteria, and thymoma prevalence was higher (16.0%) compared to the neurological ward (2.2%) and outpatient groups (1.2%). Patients in the intensive care (85.7%) and neurological ward groups (85.6%) had more comorbidities than those in the outpatient group (66.1%). The mean (±SD) CCI score was significantly higher in the intensive care (2.6 [±1.9]; *p* = 0.02) and neurological ward groups (2.7 [±2.4]; *p* < 0.001) compared to the outpatient group (1.9 [±1.9]). There was no difference in sex distribution, Ab status (AChRAb or MuSKAb), thymectomy rate, or prevalence of other autoimmune diseases among these MG groups.

The mean serum potassium level in the intensive care group at admission was lower than in the non‐intensive care patients at the latest follow‐up (3.8 [±0.4] vs. 4.0 [±0.4] mmol/L, respectively; *p* = 0.002). There was no difference in potassium levels between the patients requiring intensive care and those with no need for intensive care at their latest follow‐up (3.9 [±0.5] vs. 4.0 [±0.4] mmol/L; nonsignificant), between the intensive care group and neurological ward group (3.8 [±0.4] vs. 3.9 [±0.4] mmol/L; nonsignificant), or between the patients in intensive care due to MC (3.9 [±0.5] mmol/L) and the neurological ward group (3.9 [±0.5] vs. 3.9 [±0.4] mmol/L; nonsignificant).

Female sex and thymoma were significant predictors of increased risk for intensive care admission because of MG exacerbation or MC in binary logistic regression of risk factors including sex, age at diagnosis, onset subtype (EOMG vs. LOMG), thymectomy, thymoma and comorbidities (Table 3). In further analysis, AChR‐ab status, thymectomy delay, or age at thymectomy were not significantly associated with intensive care unit admission in pwMG (data not shown). For MC patients, thymoma was the only significant predictor for intensive care unit admission (OR 8.31, 95% CI 1.97–35.17; *p* = 0.004).

**TABLE 3:** Binary logistic regression analysis for intensive care unit admission due to myasthenia gravis exacerbation or myasthenic crisis.

Predictor	Odds ratio	95% CI		*p* value
Thymoma	4.81	1.19	19.43	0.028
Sex (female)	2.14	1.02	4.48	0.045
Thymectomy performed	1.77	0.77	4.07	NS
LOMG	1.39	0.33	5.85	NS
Age at the diagnosis of MG	1.03	0.99	1.06	NS
MG alone	0.97	0.39	2.42	NS
MG with another AD	0.72	0.30	1.72	NS

*Note*: The overall model was statistically significant (*χ* ^2^ [7] = 16.67, *p* = 0.02) and correctly predicted 90% of cases. The model overall explained 9% of the variation of intensive care unit admission (Nagelkerke *R* ^2^).

Abbreviations: AD, autoimmune disease; CI, confidence interval; MG, myasthenia gravis; LOMG, late‐onset myasthenia gravis.

Binary logistic regression, including patients from both the intensive care and outpatient groups, showed that higher CCI score was a significant predictor for intensive care unit admission due to MG exacerbation or MC (OR 1.24, 95% CI 1.03–1.49; *p* = 0.023).

We also looked at risk factors for repeated episodes of intensive care. Only four of 35 patients had more than one admission because of MG exacerbation. Two patients had >3 episodes per patient (first 10 episodes, altogether 78 days; second 9 episodes, altogether 34 days). The reasons for repetitive episodes were patient‐specific, namely, remarkable other disability and poor treatment adherence.

### Features and prognosis of intensive care episodes for MG exacerbation

Table 4 shows the clinical features of patients with intensive care for MG exacerbation or MC and their outcomes at follow‐up. The mean (SD) age at first admission was 63.4 (16.9) years. Most patients (68.6%) had their first admission within 2 years from MG diagnosis (median 5.3 months). When entering intensive care, eight patients (22.9%) were undiagnosed with MG, and three were not identified as having MG during their first episode. Infections were the most common triggering factor. Patients who did not receive rescue therapy had one of the following interventions: 1) symptomatic medication was started or increased in dose, 2) triggering factors were eliminated, 3) peroral corticosteroids were started, or 4) close follow‐up for remaining symptoms was initiated.

**TABLE 4:** Demographic data of patients with myasthenia gravis in the intensive care group and features of the intensive care.

	Patients with MG in the intensive care group
Total number of patients	35
Age at admission (SD), years	63.4 (16.9)
Time from diagnosis, median (range) months	5.3 (−50.9 –306.5)
Intensive care episode before MG diagnosis, *n* (%)	8 (22.9)
Thymectomy performed before the episode, *n* (%)	21 (60)
Time between thymectomy and the episode, median (range) months	36.9 (−61.9 –311.6)
First MG symptom, %	
Ocular	22.9
Bulbar	54.3
Weakness in limbs	14.3
No MG medication before the episode, *n* (%)	12 (34.3)
Immunosuppressive medication before the episode, *n* (%)	8 (22.9)
Triggering factor of MG exacerbation, *n* (%)^a^	
Infection	15 (42.9)
Medications	5 (14.3)
Surgery or anaesthesia	5 (14.3)
Poor compliance	2 (5.7)
Unknown	9 (25.7)
Intensive care length of stay, median (range) days	6 (1–34)
Mechanical or non‐invasive ventilation, *n* (%)	21 (51.4)
Myasthenic crisis^b^, *n* (%)	25 (71)
MG rescue therapy, *n* (%)	
Plasmapheresis or immunoadsorption	21 (60)
Intravenous immunoglobulin	3 (8.9)
Intravenous methylprednisolone	5 (14.3)
MGFA class at last follow‐up, median (range)	2 (0–4)
MGFA class when entering hospital, median (range)	4 (1–5)
Highest MGFA class in intensive care, median (range)	5 (3–5)
MGFA class at next follow‐up, median (range)	2 (0–4)
Immunosuppression enhancement after the episode, *n* (%)	18 (51.4)
MGFA class at 6 months, median (range)	2 (0–3)
Median mRS score at 6 months	2 (0–6)
Age at death (*n* = 17, during follow‐up time), mean (SD) years	75.7 (11.9)
Serum potassium level (mmol/L) at the beginning of intensive care episode, mean (SD) (all episodes, *n* = 59)	3.8 (0.4)
Highest CRP level during the episode, mean (SD) mg/L	89.8 (84.4)
In‐hospital stay, mean (SD) days	22.6 (17.0)

Abbreviations: CRP, C‐reactive protein; MG, myasthenia gravis; MGFA, Myasthenia Gravis Foundation of America, only the numeral level is used; mRS, modified Rankin Scale; SD, standard deviation.

^a^ Some patients had more than one triggering factor.

^b^ Myasthenic crisis was defined as an MG exacerbation requiring non‐invasive ventilation, intubation, or a nasogastric tube.

Patients with EOMG had shorter intensive care episodes than those with LOMG (median 4.1 days vs. 8.0 days; *p* = 0.03). Longer intensive care episode correlated with higher MGFA class during intensive care (correlation coefficient 0.0697; *p* < 0.001), extended in‐hospital stays (correlation coefficient 0.676; *p* < 0.001), and increased 6‐month mortality (correlation coefficient 0.506; *p* > 0.002). Intensive care duration correlated with mRS score before intensive care (correlation coefficient 0.345; *p* = 0.042), at hospital discharge (correlation coefficient 0.443; *p* = 0.009), and at 6‐month follow‐up (correlation coefficient 0.448; *p* = 0.013).

Higher CCI score correlated with longer in‐hospital stays (correlation coefficient 0.351; *p* = 0.039) but not with intensive care episode length, number of intensive care episodes, MGFA score, or mRS score. In addition, CCI score was not associated with MC or 6‐month mortality in the intensive care group.

The pwMG who had died within 6 months after intensive care were older at admission (median [range] 81.3 [57.6–82.5] years vs. 67.1 [12.9–84.2] years; *p* = 0.016) and had longer intensive care stay (median [range] 16 [11–34] days vs. 5 [1–18] days; *p* < 0.01). Patients with MC had longer intensive care episodes (mean [SD] 10.3 [1.6] days vs. 4.7 [1.3] days; *p* = 0.023) and higher CRP (mean [SD] 100.1 [14.7] mg/L vs. 65.1 [35.0] mg/L; *p* = 0.028) than other patients with intensive care for MG exacerbation. In a linear regression analysis of intensive care duration due to MC, disease onset type (*p* = 0.009), highest CRP level (*p* = 0.006) and mRS score at the last outpatient visit (*p* = 0.023) were significant predictors, while sex and age were not. The model was statistically significant (*R* ^2^ = 0.643, *F* (5, 19) = 6.850; *p* < 0.001). LOMG, higher mRS score and higher CRP level during intensive care were associated with longer intensive care episodes in MC patients. In‐hospital mortality for MC patients was 4.0% and 4.8% for MC patients with mechanical ventilation.

Serum potassium levels at the beginning of the intensive care episodes did not correlate with any of the clinical outcome measures including duration of intensive care or hospital stay, highest MGFA class, or mRS score at the end of hospitalisation or 6 months afterwards.

We compared the features of intensive care for *bronchial asthma* and for MG (Table 5). Patients with MG exacerbation or MC were older at intensive care unit admission, their first intensive care episode was longer, and 6‐month mortality after intensive care was twice that of asthma patients (14.3% vs. 7.7%; *p* = 0.227).

**TABLE 5:** Comparison of demographics, length of stay and mortality between all episodes of intensive care, acute exacerbation of *bronchial asthma* and myasthenia gravis exacerbation treated in intensive care during the study period.

	All intensive care episodes	Acute severe exacerbation of *bronchial asthma*	MG exacerbation or MC
Number of patients (% of the total)	106,910	142 (0.1)	35 (0.03)
Age, mean (±SD) years	61.5 (18.5)	56.0 (20.5)	63.4 (17.2)*
Female, *n* (%)	46,078 (43.1)	99 (69.7)	20 (57.1)
Median (range) duration of one intensive care episode, days	1 (0–210)	1 (0–18)	6 (1–34)**
Duration of all intensive care episodes, days (% of the total)	382,073	256 (0.07)	462 (0.1)
More than two intensive care episodes, *n* (%)	30,400 (28.4)	30 (21.1)	4 (11.4)
Mortality during intensive care episode (in 10 days), *n* (%)	8232 (7.7)	7 (4.9)	1 (2.9)
Mortality in 6 months after intensive care episode, *n* %	16,998 (15.9)	11 (7.7)	5 (14.3)

*Note*: *p* values indicating statistically significant differences between the studied subgroups of MG exacerbation and acute severe asthma are denoted with asterisks, and those not shown were insignificant.

Abbreviations: MC, myasthenic crisis; MG, myasthenia gravis; SD, standard deviation.

* *p* = 0.04.

** *p* < 0.001.

## DISCUSSION

This was a large population‐based study of a rare disease, covering a hospital district of 1.8 million people [26], and a quarter of all pwMG in Finland, of whom 6.1% required intensive care due to MC or MG exacerbation in this 14‐year period [27]. First admission occurred within a median of 5.3 months from diagnosis. Risk factors for intensive care for MG were female sex, thymoma, comorbidities and LOMG. Infection as a trigger was the reason for nearly half of the cases in intensive care due to MG exacerbation. Furthermore, MG may contribute to the need for intensive care in infectious and respiratory diseases in pwMG even without MG exacerbation. Longer intensive care period was associated with LOMG and higher mRS score in patients with MG exacerbation, while higher CRP levels were a significant factor in MC. Longer intensive care and older age correlated with mortality at 6‐month follow‐up. Six‐month mortality after intensive care for MG exacerbation was twice that of asthma exacerbation. Potassium levels were lower at intensive care admission than at follow‐up but did not affect prognosis. The MG exacerbation episodes covered only 0.03% of all intensive care periods and over 93% of pwMG did not need intensive care for MG exacerbation.

While we found that female sex was a significant risk factor, earlier studies showed conflicting results. Some found no sex difference in MC risk [7, 14], while others noted a male predominance [15, 28], or a higher risk in females [29, 30]. The bimodal sex distribution, with EOMG more prevalent in females and LOMG more prevalent in males [1], might affect these differences in findings. In our cohort, most patients needing intensive care for MG had LOMG, and most were female. Our results reflect the results of a recent large cohort study revealing a high standardised mortality ratio of 1.41 in female pwMG in Finland, as in other Nordic countries [8]. While some studies report no effect of age on MC risk [16, 31], generally age has been considered a risk factor [5, 6, 29] and our finding that LOMG was a risk factor for intensive care for MG supports this. Earlier studies report that patients with MC are slightly older than those without [7] and the mean age at admission in these studies ranges from 60 to 70 years [6, 29], which is in line with our results. Thymoma is a risk factor for MC, as also shown in earlier studies [5, 14].

Previous studies and our findings concur that older age predicts poor prognosis in MC and MG exacerbation [6, 14]. Age over 50 years at admission was identified as an independent risk factor for in‐hospital mortality, with no deaths in patients aged under 40 years among 5500 MG hospital admissions [7]. Older age correlates with prolonged mechanical ventilation need in MC, which is associated with higher mortality [6, 7, 29]. Comorbidities, which increase with age [32], are also likely to affect outcomes, as demonstrated by previous research showing that comorbidities are associated with a poorer prognosis in MG [33]. CCI score showed a positive correlation with the need for intensive care due to MG exacerbation or MC, and higher CCI scores were associated with an increased need for in‐hospital treatment for MG exacerbations [34]. Age and comorbidities may limit the treatment options for MG, and thus increase the risk of MG exacerbation. Additionally, high MGFA class before hospitalisation, infection as a trigger for exacerbation, and low vital capacity are linked to worse MC outcomes [7, 14, 19]. Among frail patients, a smaller decline in ventilation function can lead to the need for hospitalisation and ventilation support. Our findings of higher disability and infection indicators as significant outcome factors support earlier results. Most studies did not find sex to be a prognostic factor for MC [6, 7, 14], except for one study in which men had a worse prognosis [30]. Nearly half of our patients did not have enhancement of immunosuppressive medication after MG intensive care, possibly for patient‐specific reasons, including age and comorbidities.

Compared to general intensive care mortality, pwMG mortality was lower and remained the same 6 months after hospitalisation. Patients with MG exacerbation had longer intensive care episodes than *bronchial asthma* patients and all intensive care patients, possibly because of multiday rescue therapy plasma exchange. In‐hospital mortality for MC (4%) in our cohort aligns with that observed in previous studies (4%–12%) [7, 14, 20, 28, 29].

Also consistent with previous studies (13%–28% of MC cases) [6, 35, 36], one fifth of the pwMG admitted to intensive care lacked a prior MG diagnosis. This highlights the importance of suspecting MG in unclear respiratory failure cases. Compared to asthma, an autoimmune respiratory disease affecting 10% of adults in Finland [37], we found that the need for intensive care for respiratory failure is more pronounced in MG.

Limitations of this study include retrospective data collection and potential source errors from miscoding or inaccurate structural documentation of the data lake search. Manual chart review was performed only for pwMG and the data on patients with *bronchial asthma* were based solely on data lake search. The low number of intensive care patients with MC or MG exacerbation reduces the statistical power of the analyses, but considering the prevalence of the disease, our systematically collected study cohort is of a substantial size, and similar studies have not been conducted in the Nordic countries.

In conclusion, our study shows an increased risk of intensive care for MG exacerbation in patients with LOMG, several comorbidities, female sex or thymoma, occurring usually within 6 months from diagnosis. Special attention to early treatment choices should therefore be given, especially in LOMG patients. However, only 6% of pwMG were ever in need of intensive care in our hospital district, emphasising the rarity of this life‐threatening event.

## AUTHOR CONTRIBUTIONS

**Chris Myllynen:** Data curation; formal analysis; writing – original draft; investigation. **Anni Tuulasvaara:** Data curation; formal analysis; investigation; writing – original draft. **Sari Atula:** Project administration; supervision; writing – review and editing. **Sini M. Laakso:** Conceptualization; methodology; funding acquisition; project administration; writing – review and editing; supervision.

## FUNDING INFORMATION

Chris Myllynen received a grant from the Maire Taponen Foundation. Anni Tuulasvaara received research funding from the state of Finland, through Helsinki University Hospital (project code Y223230047). Sari Atula has no funding to disclose. Sini M. Laakso received research funding from the state of Finland, through Helsinki University Hospital (project code TYH2023316).

## CONFLICT OF INTEREST STATEMENT

Anni Tuulasvaara declares travel expenses from UCB Pharma. Sini Laakso declares travel expenses from UCB Pharma, lecture fees from Argenx, and advisory fees from UCB Pharma, Argenx and Alexion. The remaining authors declare no conflicts of interest.

## Data Availability

The data that support the findings of this study are available on request from the corresponding author. The data are not publicly available due to privacy or ethical restrictions.
